# Male occult breast cancer with axillary lymph node metastasis as the first manifestation: A case report and literature review

**DOI:** 10.1097/MD.0000000000009312

**Published:** 2017-12-22

**Authors:** Ruixin Xu, Jianbin Li, Yingjie Zhang, Hongbiao Jing, Youzhe Zhu

**Affiliations:** a Medicine and Life Sciences College of Shandong Academy of Medical Sciences, University of Jinan; b Department of Radiation Oncology; c Department of Pathology, Shandong Cancer Hospital Affiliated to Shandong University, Jinan, Shandong Province, China.

**Keywords:** axillary lymph node metastasis, immunohistochemical staining, male, occult breast cancer

## Abstract

**Rationale:**

Occult breast cancer (OBC) is extremely rare in males with neither symptoms in the breast nor abnormalities upon imaging examination.

**Patient concerns:**

This current case report presents a young male patient who was diagnosed with male OBC first manifesting as axillary lymph node metastasis. The physical and imaging examination showed no primary lesions in either breasts or in other organs.

**Diagnoses:**

The pathological results revealed infiltrating ductal carcinoma in the axillary lymph nodes. Immunohistochemical (IHC) staining was negative for estrogen receptor (ER), progesterone receptor (PR), cytokeratin (CK)20 and thyroid transcription factor-1 (TTF-1), positive for CK7, gross cystic disease fluid protein-15 (GCDFP-15), epithelial membrane antigen (EMA) and carcinoembryonic antigen (CEA), and suspicious positive for human epidermal receptor-2 (Her-2). On basis of IHC markers, particularly such as CK7, CK20 and GCDFP-15, and eliminating other malignancies, male OBC was identified in spite of negativity for hormone receptors.

**Interventions:**

The patient underwent left axillary lymph node dissection (ALND) but not mastectomy. After the surgery, the patient subsequently underwent chemotherapy and radiotherapy.

**Outcomes:**

The patient is currently being followed up without any signs of recurrence.

**Lessons:**

Carefully imaging examination and pathological analysis were particularly essential in the diagnosis of male OBC. The guidelines for managing male OBC default to those of female OBC and male breast cancer.

## 1 Introduction

Male breast cancer (MBC) is a rare but important condition accounting for only 1% of all breast cancer worldwide and less than 1% of all male malignancies. Male OBC is an extremely rare type of MBC that is asymptomatic in the breast and is normally detected by palpable lumps in the axilla, similar to the OBC in females. The case we reported here is a young male patient diagnosed with occult breast cancer. He had no significant medical history, and the initial clinical symptom was axillary lymph node metastasis. He underwent left axillary lymph node dissection (ALND) twice but without mastectomy. Herein, by reviewing the relevant literatures, we introduce a case of the male OBC and explore its clinical, imageological, and pathological features and the management. This study was approved by the institutional research ethics board of Shandong Cancer Hospital. Written informed consent was obtained from the patient.

## 2 Case presentation

**Figure 1:**
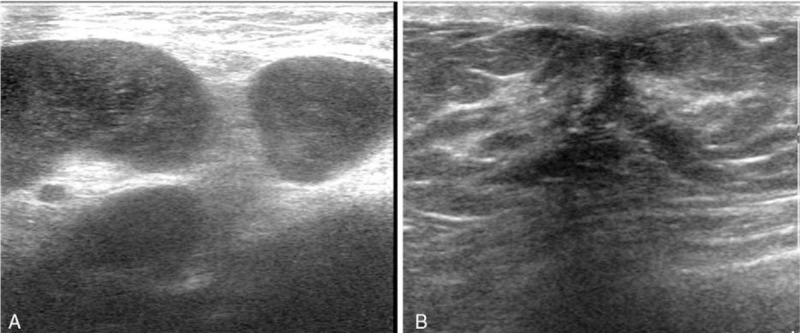
(A) Initial US revealed 3 hypoechoic swollen lymph nodes with clear boundaries; the largest mass was 4.3 cm × 2.3 cm in diameter. (B) Initial left breast US revealed hyperplasia of the mammary gland with a maximum scope of 18 mm × 18 mm.

**Figure 2:**
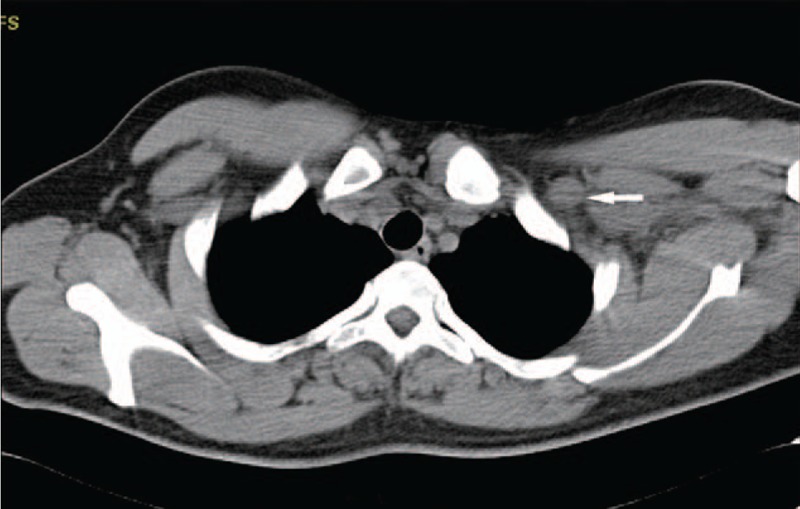
Chest CT showed an enlarged lymph node in the infraclavicular region (arrow) after the first surgery. CT = computed tomography.

**Figure 3:**
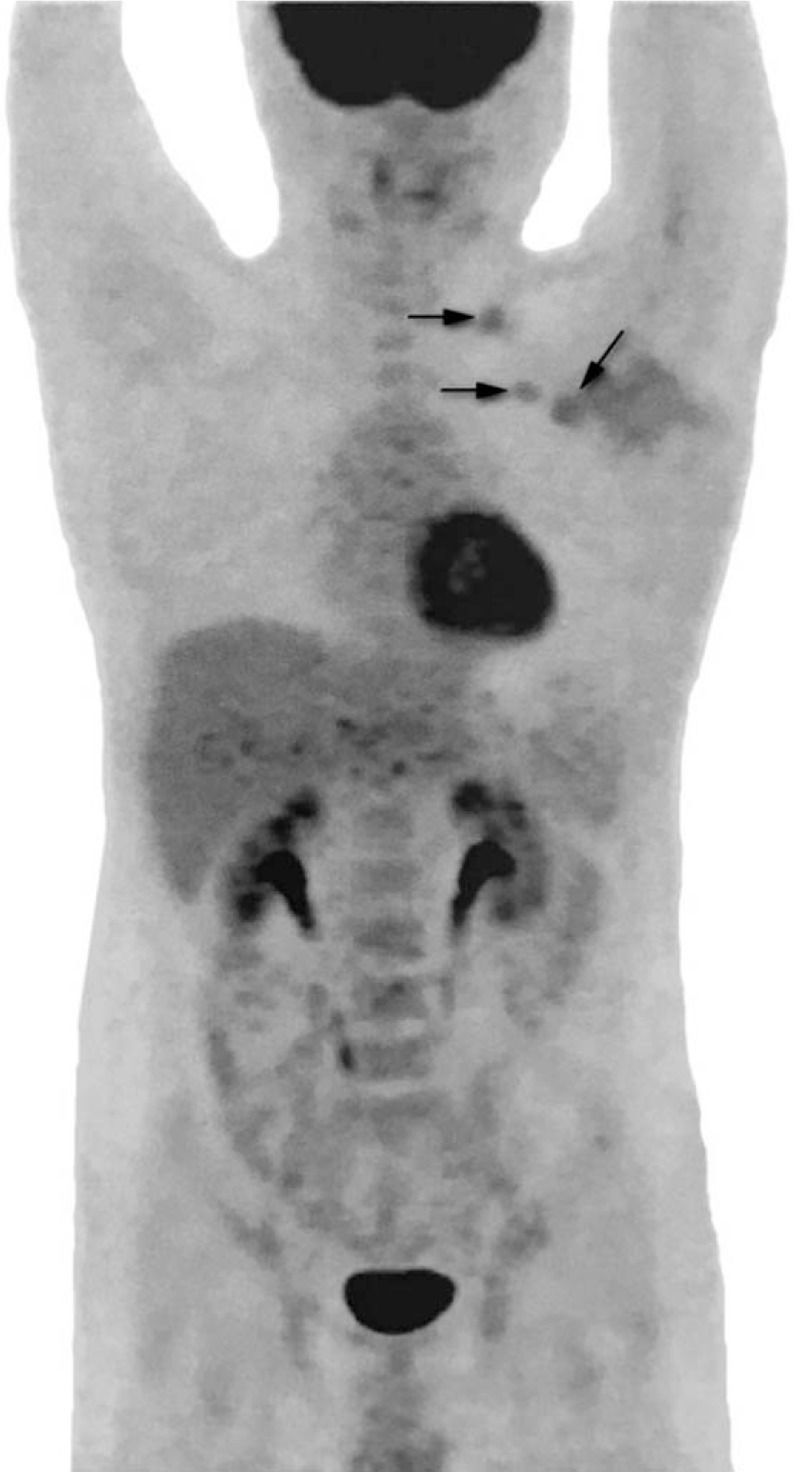
PET/CT scan of the entire body showed several lesions with increased uptake in the left axillary and infraclavicular regions (arrows) after the first surgery. PET/CT = positron emission tomography/computed tomography.

The patient, a 29-year-old male, discovered a painless half-peanut-size nodule in his left axilla in April 2014. The patient was not concerned because of his busy daily work schedule. The size of the nodule gradually increased in the following year. In March 2016, he went to a local hospital near his home for a medical checkup and exhibiting 2 walnut-sized palpable masses. Ultrasonographic (US) examination revealed 3 hypoechoic solid masses with clear boundaries in the left axillary cavity (Fig. 1); the largest mass was 4.3 cm × 2.3 cm in diameter, and the first-stage diagnosis was “The solid masses in the left axilla.” He then went to the surgical department for further examination and treatment. Physical examination in the left axilla showed 2 rigid, mobile, painless masses measuring approximately 5 cm in diameter, and the skin covering the masses was normal. After admission to the hospital, all tumor markers, such as serum carcinoembryonic antigen (CEA), neuron-specific enolase (NSE), alpha fetoprotein (AFP), prostate specific antigen (PSA), cytokeratin 19 fragment (Cyfra21–1), carbohydrate antigen 19–9 (CA19–9), and carbohydrate antigen 72–4 (CA72–4), were within normal ranges. Both routine hematological and biochemical parameters were normal. On March 16, 2016, the patient underwent his first surgery for axillary neoplasm resection and left axillary lymph node dissection (ALND). The pathological report of the local hospital (with consultation from the superior hospital expert) stated that only metastatic lymph nodes were found in the left axilla with no suspicious lesions in the resected specimen. A poorly differentiated carcinoma was found in the lymph nodes (infiltrating ductal carcinoma, IDC; 14 out of 15 dissected axillary lymph nodes showed metastasis), and according to IHC staining, the probability that it metastasized from breast cancer could not be ruled out. The IHC results were positive for cytokeratin (CK), gross cystic disease fluid protein 15 (GCDFP-15) and human epidermal receptor 2 (Her-2) (++) and negative for estrogen receptor (ER), progesterone receptor (PR), CK5/6, CK20, caudal-related homeobox transcription factor 2 (CDX-2), P63, thyroid transcription factor 1 (TTF-1), synaptophysin (Syn), chromogranin A (CgA), and S100. A chest computed tomography (CT) scan was performed in the local hospital 8 days after the operation, and multiple enlarged lymph nodes were shown in the left axillary and infraclavicular regions (Fig. 2). The patient did not receive further treatment and was discharged without any complications on March 26, 2016. Two days later, he reported to a superior hospital and underwent a positron emission tomography with integrated computed tomography (PET-CT) scan of the entire body. The scan showed several lesions with increased uptake in the left axillary and infraclavicular regions (Fig. 3), but no malignant lesions in other organs, especially in the mammary glands. The US examination on the same day also showed no lesions on the breast, and in addition, there was no manifestation of accessory breast or ectopic breast in the axilla. Mammography was strongly recommended but the patient declined.

**Figure 4:**
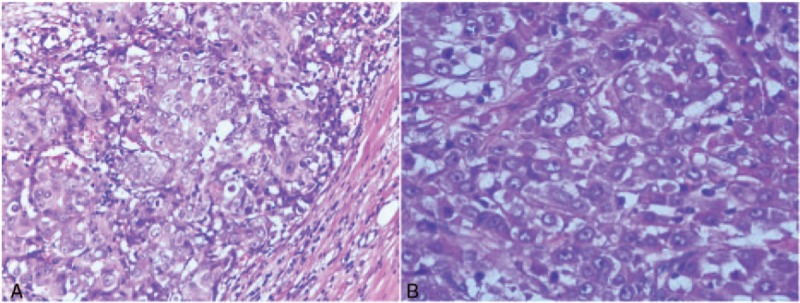
Postoperative HE (original magnification ×100 for A and ×200 for B) staining. HE = hematoxylin and eosin.

**Figure 5:**
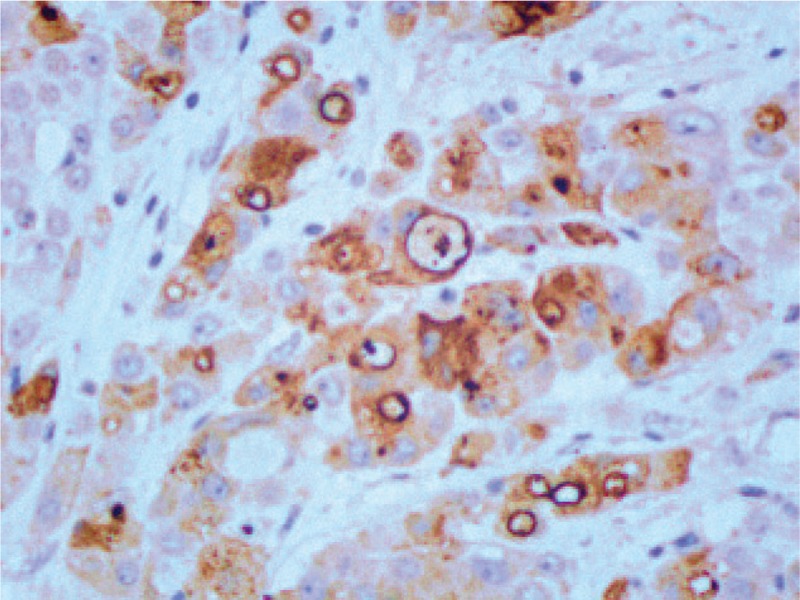
IHC staining (original magnification ×200) showing positivity for GCDFP-15. GCDFP-15 = gross cystic disease fluid protein-15, IHC = immunohistochemical.

**Figure 6:**
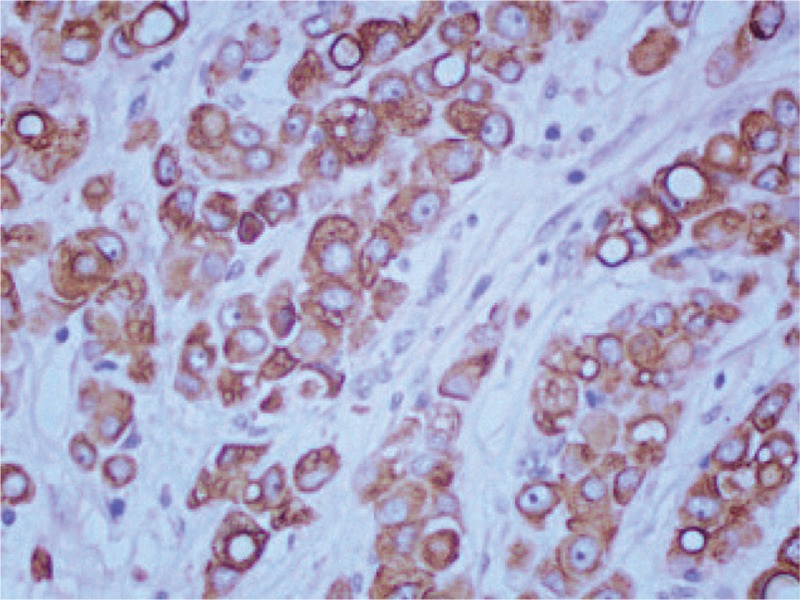
IHC staining (original magnification ×200) showing positivity for CK7. CK = cytokeratin, IHC = immunohistochemical.

**Figure 7:**
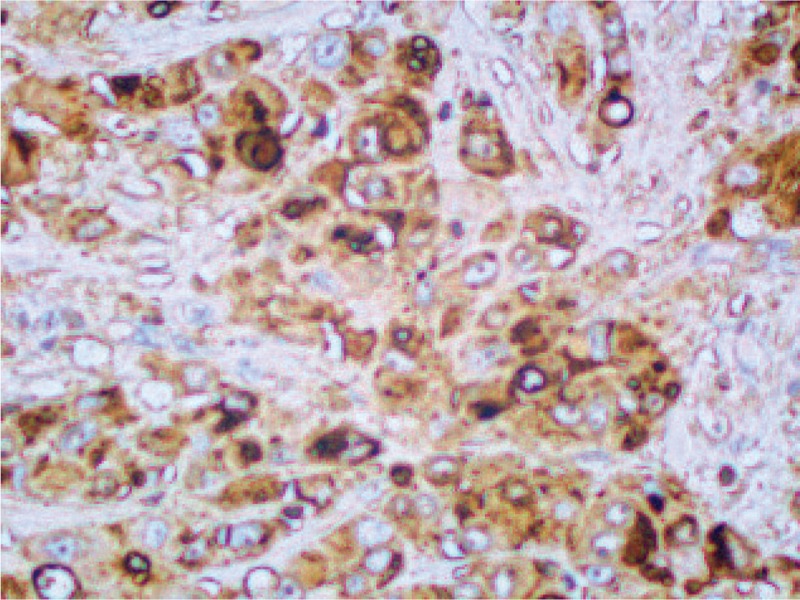
IHC staining (original magnification ×200) showing positivity for EMA. EMA = epithelial membrane antigen, IHC = immunohistochemical.

**Figure 8:**
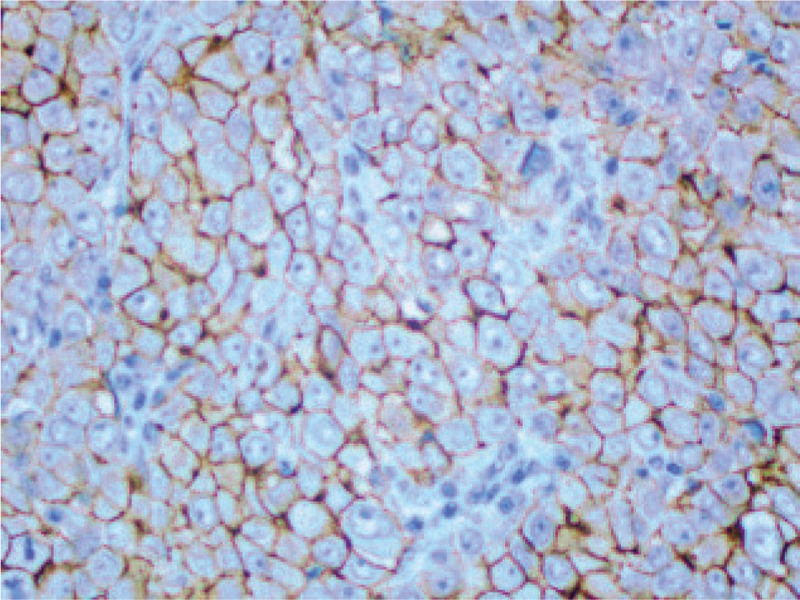
IHC staining (original magnification ×200) showing suspicious positivity for Her-2. Her-2 = human epidermal receptor-2, IHC = immunohistochemical.

On April 4, 2016, the patient presented to our hospital. He had no history of disease other than an appendectomy 3 years ago, and no history of medication, particularly hormonal drugs. He had an 8 pack-years smoking history and was a nondrinker. His mother had died of gastric cancer and his father of heart attack. In a physical examination, a 6-cm surgical scar was found on the left axilla; around the scar, several palpable rigid nodules were found under the skin. No symptoms were found in either breasts or in other organs. Considering all the results above, pathologists and oncologists eventually concluded that this was a case of male OBC. We recommended a modified radical mastectomy, but he refused the mastectomy except for ALND. On April 7, 2016, he underwent his second surgery. Histological examination of paraffin sections with hematoxylin and eosin (HE) staining showed that only the left axillary (levels I and II) and infraclavicular (level III) lymph nodes were invaded by IDC, but no other lesions were found in the rest of the specimen. The tumor node metastasis (TNM) classification was T0N3aM0 Stage IIIC, according to 7th American Joint Committee on Cancer (AJCC) staging system of breast cancer. Histological examination showed that the structure of the lymph node was destroyed, and tumor cells with no clear boundaries were arranged in a prominent massive, diffusive, and nesting pattern. They were polygonal in shape, with abundant pink cytoplasm and large oval nuclei (Figs. 4 and 5). IHC staining (Figs. 6–8) showed positivity for CK7, GCDFP-15, EMA, and CEA, suspicious positivity for Her-2 (++), and negativity for ER, PR, GATA-binding protein-3 (GATA-3), CK20, and mammaglobin. The postoperative pathological report again revealed carcinoma in the left axillary and infraclavicular lymph nodes that metastasized from the breast. Because of the suspicious positivity for Her-2, the fluorescent in situ hybridization (FISH) method was strongly recommended but the patient declined because of inability to afford trastuzumab. After the surgery, the patient subsequently underwent adjuvant chemotherapy (AT scheme: adriamycin 40 mg day 1–2; docetaxel 130 mg day 1, every 3 weeks for 6 cycles). The treatment went smoothly, and the patient had good tolerance. Postoperative adjuvant radiation therapy on his remaining breast was delayed due to poor healing of the incision (wound infection). He told us that the wound healed in December 2016 and that he underwent radiation therapy in February 2017. The patient is currently being followed up without any signs of recurrence.

## 3 Discussion

In 1907, Halsted first described 2 patients with extensive carcinomatous involvement of the axilla by OBC.^[1]^ Approximately 0.3% to 1.0% of breast cancer cases are OBC.^[2,3]^ Breast cancer is rare in men, accounting for less than 1% of all malignances in men and only 1% of all cases of breast carcinoma.^[4,5]^ The global incidence rates of male breast cancer vary widely by location and ethnicity.^[6]^ MBC has a unimodal age distribution with a peak incidence at 71 years, differing from the bimodal distribution in female breast cancer (FBC).^[7]^ Fentiman et al^[4]^ reported that the majority of male breast cancers, including this case, are the invasive ductal type (85–95%), followed by ductal carcinoma in situ (10%), and therefore, the present case is among the majority. Male OBC is an extremely rare type of MBC or OBC. It was asymptomatic in the breast and usually discovered due to symptoms of metastasis in the axillary area, supraclavicular fossa, or infraclavicular fossa.^[8–11]^ This is the sixth case report of male OBC in the literature. The characteristics of these cases are summarized in Table 1. Among these reported cases, the male OBC occurred from age 40 to 78, and thus, our case was the youngest.

**Table 1:**
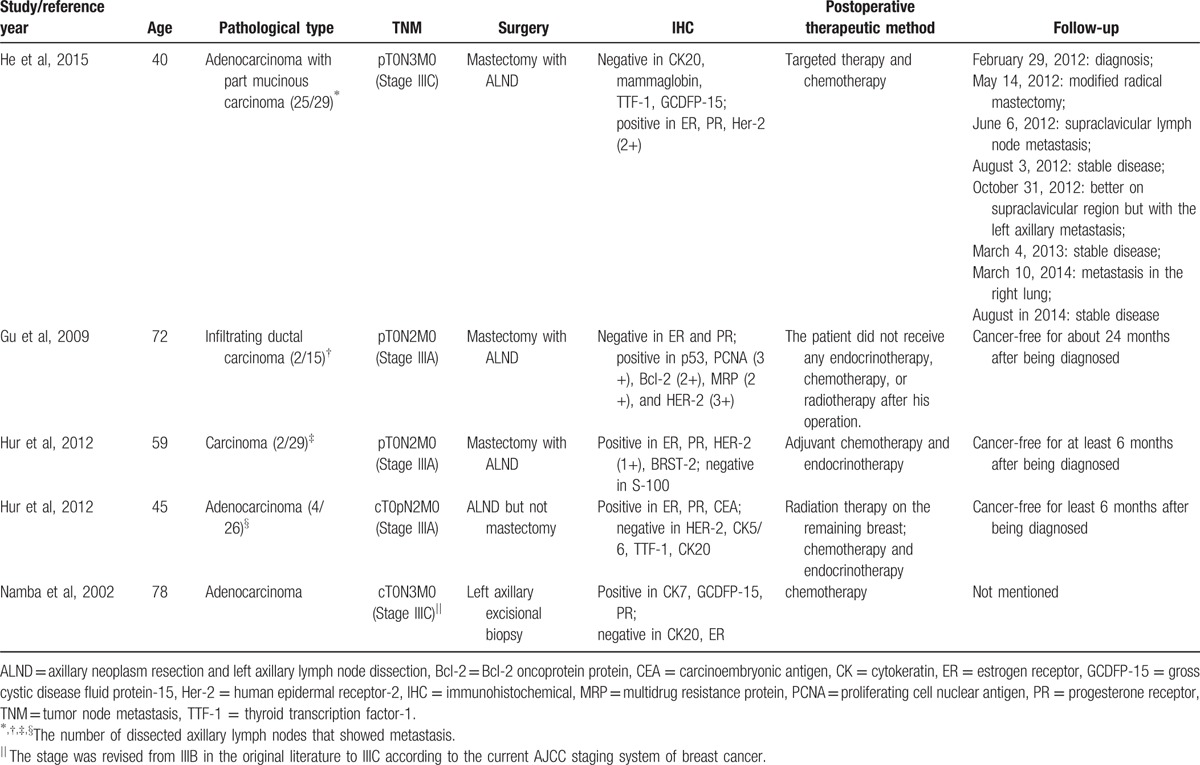
Summary of the cases reported on the male occult breast cancer.

Clinically, when only swollen axillary lymph nodes without a primary lesion are found, the first step is to clarify whether they are benign or malignant and then to confirm the original organ.^[8]^ The most commonly reported palpable axillary masses are metastatic lymph nodes from breast cancer,^[12,13]^ and followed by other malignancies including lung cancer, digestive tract cancer, hematological tumor, thyroid cancer, ovarian cancer, and accessory breast cancer. To avoid misdiagnosis, a comprehensive and systematic physical and imaging examination should be carried out to identify the primary origin. The absence of mammography or MRI of the breast is a drawback of our case report. It has been reported that magnetic resonance imaging (MRI) is a powerful tool to discover occult lesions that are not found by physical examination or routine imaging therapies such as CT and US.^[14–16]^

Our case is similar to the 5 reported cases diagnosed with male OBC. The cases had the following in common. First, the symptom was a painless palpable mass in the axilla. Second, both physical and imaging examinations revealed no primary lesions on the breast or other organs. Lastly, the tumor in the axillary lymph node was pathologically confirmed to have metastasized from the breast according to IHC. It has been concluded that the frequency of ER positivity in MBC is higher than that in female breast cancer (FBC), with more than 90% of tumors being positive for ER, and more than 80% positive for PR.^[7,11,17]^ Therefore, the presence of both ER and PR plays a crucial role in determining whether the primary disease behind the axillary metastatic lymph node is breast cancer. In addition, even if both ER and PR are negative, breast cancer cannot be eliminated as a possible cause.^[11]^ The case of male OBC reported by Gu et al,^[9]^ as in our case, showed no expression of ER and PR. The final diagnosis was confirmed after consulting a number of pathologists and performing a series of examinations for differential diagnosis. The importance of carefully histological examination and IHC staining was particularly emphasized in the diagnosis of OBC. In diagnosing metastatic breast cancer, CK7 and CK20 are the most common IHC markers, and the differential diagnosis scope can be narrowed based on these markers. It has been reported that CK7 is usually positive and CK20 is usually negative in breast cancer. CK is expressed in more than 90% of breast cancer, whereas CK20 is mainly expressed in gastrointestinal tumors.^[18]^ Another IHC marker of great importance is GCDFP-15,^[19]^ which has a positive predictive value and a specificity of 90% in the diagnosis of breast cancer.^[20]^ On the basis of all the results above, the patient was diagnosed with male OBC.

A standard surgical treatment for MBC is modified radical mastectomy.^[11]^ By reviewing the 5 reported cases, we found that 4 in 5 patients underwent surgery. In the 4 patients, one of them underwent ALND only, whereas the others underwent both mastectomy and ALND. Walker et al^[21]^ compared the 10-year overall survival (OS) of 596 OBC patients who underwent different treatments. The results showed that patients who underwent Mast (mastectomy plus ALND with or without postmastectomy radiation) or BCT (breast-conserving therapy with ALND and radiation) (n = 470) had a 10-year OS rate of 64.9% compared with 58.5% for patients who underwent ALND only (n = 126; *P* = .02) and 47.5% for patients who underwent observation only (n = 94; *P* = .04). The 10-year cause-specific survival (CSS) rate was 75.7% for patients who underwent BCT versus 73.9% for patients who underwent Mast (*P* = .55). Another study reported similar results.^[22]^ We inferred that the postoperative radiation is crucial for OBC patients with axillary metastasis who undergo ALND only, similar to our case. To date, however, there have been no reports, on the therapeutic methods for male OBC.^[11]^ Systematic treatment guidelines recommended for MBC default to postmenopausal FBC,^[6]^ such as surgery combined with chemotherapy, radiotherapy, endocrine therapy, or targeted therapy. Therefore, we can infer that these guidelines are also used for male OBC. The patient in our case report had undergone chemotherapy and radiotherapy after surgery.

The axillary lymph node status has been accepted as one of the established factors that guides risk allocation in cases of OBC.^[23]^ Studies have suggested that extensive lymph node involvement is an additional unfavorable prognostic factor for patients with OBC.^[21,24,25]^ We suspected that our case might have a poor outcome because of the poor axillary lymph node status. Generally, breast cancer patients with poorly differentiated carcinoma had unfavorable prognoses, although the opposite is true for OBC patients. The majority of prognosis studies have shown that the OS of OBC is slightly better than that of breast cancer at a comparable nodal stage.^[22,26–28]^ Male OBC may have its own characteristics that differ from female OBC or MBC, but little is known about male OBC due to its particularly low incidence rate. Multicentric and worldwide research of the biological features and classification of male OBC and further case registration are needed to acquire more data to guide clinical diagnosis and treatment.
